# Tuberculosis Contact Screening and Isoniazid Preventive Therapy in a South Indian District: Operational Issues for Programmatic Consideration

**DOI:** 10.1371/journal.pone.0022500

**Published:** 2011-07-22

**Authors:** Madhavi Pothukuchi, Sharath Burugina Nagaraja, Santosha Kelamane, Srinath Satyanarayana, Shashidhar, Sai Babu, Puneet Dewan, Fraser Wares

**Affiliations:** 1 Department of Community Medicine, Siddhartha Medical College, Vijayawada, Andhra Pradesh, India; 2 WHO-RNTCP Technical Assistance Project, Andhra Pradesh, India; 3 Center for Operations Research, International Union against Tuberculosis and Lung Diseases, New Delhi, India; 4 District Tuberculosis Centre, Machilipatnam, Krishna District, Andhra Pradesh, India; 5 State TB Office, Directorate of Health, Government of Andhra Pradesh, Hyderabad, India; 6 Office of the WHO Representative in India, World Health Organization, New Delhi, India; University of Stellenbosch, South Africa

## Abstract

**Background:**

Under India's Revised National Tuberculosis Control Programme (RNTCP), all household contacts of sputum smear positive Pulmonary Tuberculosis (PTB) patients are screened for TB. In the absence of active TB disease, household contacts aged <6 years are eligible for Isoniazid Preventive Therapy (IPT) (5 milligrams/kilogram body weight/day) for 6 months.

**Objectives:**

To estimate the number of household contacts aged <6 years, of sputum smear positive PTB patients registered for treatment under RNTCP from April to June'2008 in Krishna District, to assess the extent to which they are screened for TB disease and in its absence initiated on IPT.

**Methods:**

A cross sectional study was conducted. Households of all smear positive PTB cases (n = 848) registered for treatment from April to June'2008 were included. Data on the number of household contacts aged <6 years, the extent to which they were screened for TB disease, and the status of initiation of IPT, was collected.

**Results:**

Households of 825 (97%) patients were visited, and 172 household contacts aged <6 years were identified. Of them, 116 (67%) were evaluated for TB disease; none were found to be TB diseased and 97 (84%) contacts were initiated on IPT and 19 (16%) contacts were not initiated on IPT due to shortage of INH tablets in peripheral health centers. The reasons for non-evaluation of the remaining eligible children (n = 56, 33%) include no home visit by the health staff in 25 contacts, home visit done but not evaluated in 31 contacts. House-hold contacts in rural areas were less likely to be evaluated and initiated on IPT [risk ratio 6.65 (95% CI; 3.06–14.42)].

**Conclusion:**

Contact screening and IPT implementation under routine programmatic conditions is sub-optimal. There is an urgent need to sensitize all concerned programme staff on its importance and establishment of mechanisms for rigorous monitoring.

## Introduction

Young children living in close contact with a case of smear-positive pulmonary TB (PTB) are at higher risk of *Mycobacterium tuberculosis* infection and TB disease [1]. The World Health Organization (WHO) recommends screening household contacts of an infectious source case to identify children with TB disease, and enable their prompt treatment, and to provide children who do not have TB disease with Isoniazid Preventive Treatment (IPT). Daily Isoniazid (INH) for at least 6 months given as preventive therapy to young children has been shown to greatly reduce the likelihood of the child developing TB disease during childhood [2], [3].

Although in many countries, the National TB Control Programmes (NTP) recommend contact screening and IPT, in particular for children under 6 years of age, preventive treatment of children often is not accorded high priority and is not generally viewed as an essential element of TB control activities. There is limited information on the implementation of this component of NTP activities in the field and the operational challenges associated with its implementation in different countries have not been widely investigated [4].

As per India's Revised National Tuberculosis Control Programme (RNTCP) guidelines, all household contacts of sputum smear positive pulmonary tuberculosis (PTB) patients are to be screened for TB disease. In the absence of active TB disease, contacts aged less than 6 years are eligible for 6 months of daily IPT (with 5 mgs INH per Kg body weight per day) [5]. The operational research study reported on was undertaken to estimate the number of household contacts (aged <6 years) of sputum smear PTB patients in a patient cohort registered for treatment in a district of the southern Indian state of Andhra Pradesh, numbers screened for TB disease and in the absence of TB disease, numbers initiated on IPT under routine programmatic conditions.

## Methods

### Setting

The study was conducted in Krishna District of Andhra Pradesh State. The district has a population of 4.6 million (2008 data). The predominant occupation of people in this district is agriculture, and people tend in live in nuclear families with an average size of the family to be 4–5 persons. People who do not own land often migrate seasonally in search of employment. The district has 9 RNTCP Tuberculosis Units (A TB Unit covers a population of approximately 500,000 and acts as a sub-district supervisory unit under RNTCP) and 45 designated microscopy centres. All diagnosed TB patients initiated on treatment in these Tuberculosis Units are registered in a TB register, which is maintained by a paramedical supervisory staff (also called as Senior Treatment Supervisor).

### Case definitions, contact screening and IPT under RNTCP

A sputum smear positive PTB patient is defined as “A patient with at least 2 initial sputum smear examinations (direct smear microscopy) positive for acid-fast bacilli (AFB); Or: A patient with one sputum examination positive for AFB and radiographic abnormalities consistent with active pulmonary TB as determined by the treating Medical Officer (MO); Or: A patient with one sputum specimen positive for AFB and culture positive for M. tuberculosis”.

Household members of a sputum positive TB patient were defined as all persons who have food from the same kitchen as that of the sputum positive TB patient. A household contact for this study was defined as “a child aged less than 6 years of age who lives or has lived (irrespective of the duration) within the household of the smear positive PTB patient during the course of his/her disease (after the onset of symptoms) and till the end of treatment”.

Under RNTCP, after the diagnosis of sputum smear positive PTB patient, in addition to the counseling provided by the medical officer, a paramedical health worker is expected to visit the household of the patient, provide information to the patient and their family about TB disease and its treatment, undertake a symptom screening of all household contacts, refer all contacts aged <6 years to the nearest health facility for further evaluation by a medical officer. History of cough and/or fever and/or weight loss and/or no weight gain constitutes a TB suspect. In the absence of any of these symptoms, no further investigations are done on the child contact and are given INH prophylaxis., and ensure initiation of IPT in those with no evidence of TB disease [5].

### Study design, study population, and sampling

This was a cross sectional study of all household contacts aged <6years of a cohort of sputum smear positive PTB patients (N = 848) registered under RNTCP from April to June 2008. The study was conducted during October to December 2008.

### Study variables, source of information and data collection

Trained paramedical workers visited the households of all 848 sputum smear positive PTB patients and collected information from the head of the household on the number of household contacts aged <6 years, whether they were screened for TB disease, the number diagnosed with TB disease and in its absence initiation on IPT, and whether the household was in an urban or rural locality. Approximately 10% of the cases were cross verified by the principal investigators to assess the validity of the information collected by the trained paramedical workers.

### Data entry and analysis

Data were entered into a structured format created in Microsoft Excel, cross verified and compared for consistency. All variables are described by proportions.

### Ethics issues

This study was reviewed and approved by the institutional ethical committee of the Siddhartha Medical College, Vijayawada, Andhra Pradesh and by the Department of Health Services, Government of Andhra Pradesh. Persons included in the study were household contacts of those patients notified to the RNTCP as sputum smear-positive PTB cases. Contacting the households of these patients was considered a routine public health function of the RNTCP and offered immediate benefit to patients and the community; and as the study was being undertaken by the local programme managers whose job was to routinely evaluate the services provided, individual patient consent was therefore determined to be unnecessary. All eligible household contacts that were symptomatic, or not screened for TB disease, or were not on IPT, were referred for initiation of appropriate care.

## Results

Of the 848 sputum positive PTB patients registered for treatment, the study investigators were able to contact the households of 825 (97.2%) patients. The households of remaining 23 TB patients could not be contacted, as they had migrated out of the study area. Out of the 825 sputum smear positive cases, households of 84 sputum smear positive TB patients were visited by the principal investigators and used the same study instrument to assess whether the information gathered is correct or not. Discrepancy was noted in ∼3% of the data collected when all variables were put together.

**Figure 1:**
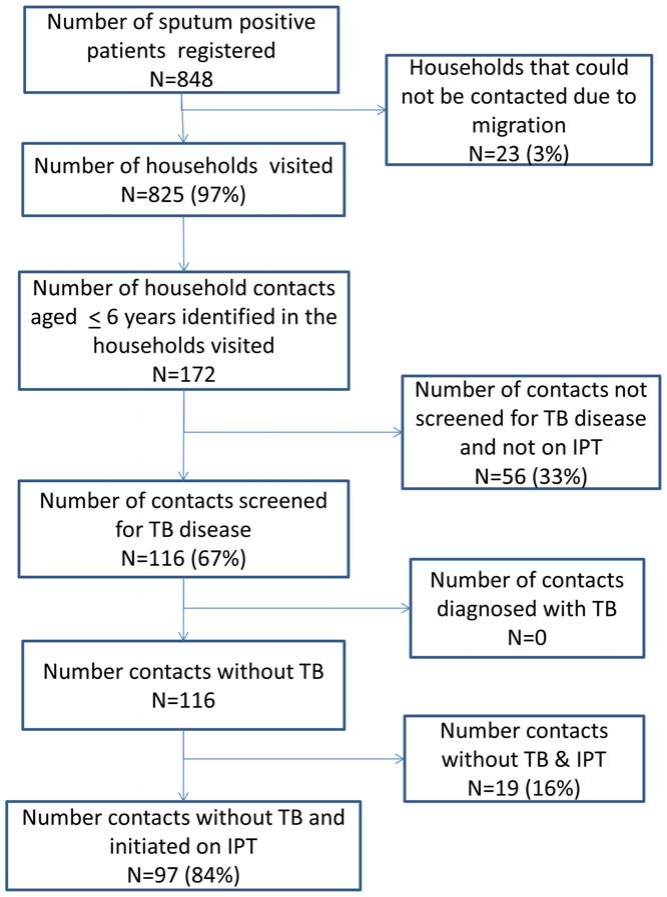
Contact Screening and IPT provision to childhood contacts of sputum smear positive PTB Patients, Krishna District, South India.

The 825 TB patients included 628 (76%) men and 197 (24%) women. The mean age of the smear positive TB patients was 46 years (Standard deviation 15.8 years).There were a total of 172 children <6 years of age who were determined to be household contacts (**Table 1**). The ratio of smear positive PTB patients to child contacts below 6 years was found to be 5∶1. Of the total contacts, 116 (67.5%) contacts were screened for TB disease, none were found to have TB disease, and 97 (84%) of them were on IPT (**Figure 1**). Out of the 97 children who were initiated on IPT, 83 (85.5%) children were consuming Isoniazid tablets regularly as outlined under the programme.

**Table 1:** Distribution of household contacts (aged <6 years) of sputum smear positive PTB patients.

Number of Children aged <6 years per Household	Number of House holds		Total number of children
	N	%	N = 172
0	653	82%	0
1	131	16%	131
2	10	1%	20
3	7	1%	21

The reasons given for not initiating IPT in 19 screened children was due to shortages of INH tablets at the peripheral health centers. Reasons for not screening 56 contacts included no home visits made by health staff (31 cases), and home visits done but no information for screening the contacts were provided to the family members by the visited health workers (25 cases). Household contacts in rural localities were less likely to be screened for TB disease and/or initiated on IPT compared to those in urban localities (**Table 2**).

**Table 2:** Urban and rural differences in childhood (aged <6 years) contact screening and IPT initiation.

Settings	No screening and/or No IPT		Screening+IPT			
	n	%	n	%	Total	Risk ratio
Rural	69	63%	40	37%	109	6.65(95% CI; 3.06–14.42)
Urban	6	10%	57	90%	63	
Total	75	44%	97	56%	172	

## Discussion

This study is one of the few studies conducted under the Indian NTP which has examined the implementation of contact screening and provision of IPT. Like previous studies conducted elsewhere and in India, the study findings show sub-optimal implementation of contact screening and IPT [6]–[11]. There appears to be a lack of importance given the implementation of these activities under the NTPs even though it is one of the most cost effective approaches to prevent childhood TB disease, especially in resource limited settings [12].

As per the WHO Guidance for National Tuberculosis Programmes on the Management of Tuberculosis in Children, in order to implement IPT and monitor this activity efficiently, all health staff must be adequately trained and sensitized on the importance of contact screening and they must be effective in passing on this information to the community. In addition, the necessary tools for diagnosis of tuberculosis such as chest radiography and tuberculin must be made available in peripheral health facilities along with Isoniazid tablets, in appropriate doses and adequate quantities. The information on the number of child contacts, their screening for tuberculosis and IPT provision must be documented on the TB treatment cards. This information must be periodically evaluated by the local programme managers to assess for the adequacy of its implementation [3]. The study provides information to fill the gaps in the programmatic processes of RNTCP for implementing this guideline efficiently.

First, the study provides an estimate of the ratio of the eligible children to sputum smear positive TB patients. The ratio of approximately 1 child contact for every 5 smear positive PTB patients are helpful in estimating the probable number of child contacts below 6 years in a district. In the absence of previous such estimates from Indian NTP, it is very difficult to comment on whether this ratio is appropriate or not. The mean age of the sputum positive TB patients in this area is 46 years, which is similar to the mean age of the sputum smear positive TB patients in southern India. This higher mean age may partly explain why a large number of index cases had no children less than 6 years of age. The estimates of the ratio may be used by the programme managers in sensitizing the peripheral health workers to be more proactive in seeking out for contacts during the initial and subsequent home visits. It also helps in planning the procurement and distribution of drugs required to provide IPT so that there are no shortages in the field.

Second, initial home visit by the paramedical health workers to identify and refer eligible children for TB screening and IPT is not being followed. This may be either due to lack of monitoring or deficiencies in paramedical workers' knowledge pertaining to the importance of this activity. The implementation of contact screening and IPT appears to be challenging particularly in rural areas as compared to urban areas. Distance to the nearest health facility, knowledge of the risk of transmission, patient and family counseling on screening, acceptance and adherence to IPT, inadequate monitoring of the implementation of this guideline are known barriers for implementing contact screening and IPT [4], [11]. These may all have to be addressed to ensure the effective implementation of screening and IPT.

Finally, and most importantly, it was somewhat surprising that no TB cases were detected amongst the screened children despite the knowledge that a significant proportion of childhood contacts may have TB disease (up to 12% in Indian settings) at the time of diagnosis of TB in adult member of the household [13], [14]. This may relate to the challenges in diagnosing TB disease in children under routine programmatic conditions despite the availability of guidelines and diagnostic algorithms under RNTCP for diagnosing and treating TB patients in children [5], [15], [ and 16]. It is very well known that the tuberculin required for tuberculin skin test and facilities for chest radiography are not often in peripheral health facilities. The extent to which the non-availability of these tests is contributing for relatively fewer TB diagnoses among children is not known.

### Limitations

First, this study was conducted in one of the most populated districts of south India. Whether the findings in this district are representative of implementation of contact screening and IPT in whole of the country is unknown. However the operational issues identified by the study and a similar study in the state of Tamil Nadu in south India [11], stimulated discussions during the routine review meetings of NTP managers in Andhra Pradesh and provided useful guidance on assessing the situations in other parts of the state and the planning of corrective measures.

Second, all children who were not screened for TB and/or were not initiated on IPT were subsequently, referred for TB screening and initiating IPT as an outcome of the study procedure. The documentation of the outcome of this referral was not part of this operational research study and hence we did not collect this information on the outcome of this referral systematically. Had we collected the outcome of this referral, we would have been able to provide further information on what proportion of the children who were not initiated on treatment was indeed having TB.

Finally, contact screening and IPT is said to be particularly useful in case of HIV positive contacts [2]. We however did not collect information on the HIV status of contacts, and hence could not assess the status of provision of services to the contacts who were HIV positive.

### Conclusions and recommendations

Contact screening under routine programmatic conditions is sub-optimal as one-third of the eligible contacts were not subjected to screening in one of the largest districts of south India. Programme managers and field level staff need to be more aware of the importance of implementation of contact screening and provision of IPT in young children. There is a need for introduction of rigorous monitoring of this component of the NTP to help assist the local programme managers, especially in rural areas.

## References

[1] Nelson LJ, Wells CD (2004). Global epidemiology of childhood tuberculosis.. Int J Tuberc Lung Dis.

[2] Stop TB Partnership Childhood TB Subgroup (2007). Chapter 4: childhood contact screening and management.. Int J Tuberc Lung Dis.

[3] World Health Organisation, Geneva (2006).

[4] World Health Organisation, Geneva (2007). A Research Agenda for Childhood Tuberculosis..

[5] Central Tuberculosis Division (2005). Technical and Operational Guidelines for Tuberculosis Control, Revised National Tuberculosis Control Programme..

[6] van ZS, Marais BJ, Hesseling AC, Gie RP, Beyers N (2006). Adherence to anti-tuberculosis chemoprophylaxis and treatment in children.. Int J Tuberc Lung Dis.

[7] Marais BJ, van ZS, Schaaf HS, van AM, Gie RP (2006). Adherence to isoniazid preventive chemotherapy: a prospective community based study.. Arch Dis Child.

[8] Zachariah R, Spielmann MP, Harries AD, Gomani P, Graham SM (2003). Passive versus active tuberculosis case finding and isoniazid preventive therapy among household contacts in a rural district of Malawi.. Int J Tuberc Lung Dis.

[9] Van Wyk SS, Hamade H, Hesseling AC, Beyers N, Enarson DA (2010). Recording isoniazid preventive therapy delivery to children: operational challenges.. Int J Tuberc Lung Dis.

[10] Nguyen TH, Odermatt P, Slesak G, Barennes H (2009). Risk of latent tuberculosis infection in children living in households with tuberculosis patients: a cross sectional survey in remote northern Lao People's Democratic Republic.. BMC Infect Dis.

[11] Banu Rekha VV, Jagarajamma K, Wares F, Chandrasekaran V, Swaminathan S (2009). Contact screening and chemoprophylaxis in India's Revised Tuberculosis Control Programme: a situational analysis.. Int J Tuberc Lung Dis.

[12] Nair N (2001). Childhood tuberculosis: public health and contact tracing.. Paediatr Respir Rev.

[13] Ramakrishanan CV, Adrews RH, Devadatta S, Fox W, Radhakrisha S (1961). Prevalence and early attack rate of tuberculosis among close family contacts of tuberculous patients in South India under domiciliary treatment with isoniazid plus PAS or isoniazid alone.. Bull World Health Organ.

[14] Sinfield R, Nyirenda M, Haves S, Molyneux EM, Graham SM (2006). Risk factors for TB infection and disease in young childhood contacts in Malawi.. Ann Trop Paediatr.

[15] Central Tuberculosis Division (2006). Guidelines for use of Pediatric Patient Wise boxes under the Revised National Tuberculosis Control Programme..

[16] Chauhan LS, Arora VK (2004). Management of pediatric tuberculosis under the Revised National Tuberculosis Control Program (RNTCP).. Indian Pediatr.

